# Acitretin chemoprophylaxis in Muir-Torre syndrome: A case report with longitudinal follow-up

**DOI:** 10.1016/j.jdcr.2024.07.017

**Published:** 2024-08-05

**Authors:** Andrew X. Tran, Christine Jaworsky

**Affiliations:** Department of Dermatology, Case Western Reserve University School of Medicine and MetroHealth System, Cleveland, Ohio

**Keywords:** case report, colon carcinoma, Muir-Torre syndrome, retinoid, sebaceous carcinoma

## Introduction

Muir-Torre syndrome (MTS) is a phenotypic variant of hereditary nonpolyposis colorectal carcinoma caused by mutations in DNA mismatch repair genes. This syndrome is characterized by the presence of sebaceous neoplasms arising in association with visceral tumors. Retinoids have been reported as chemoprophylactic agents for cutaneous and gastrointestinal malignancies in MTS. Herein, we present a patient with MTS who is in remission with her gastrointestinal carcinomas and maintains stability with her cutaneous neoplasms while on acitretin chemoprophylaxis for 11 years.

## Case report

**Fig 1:**
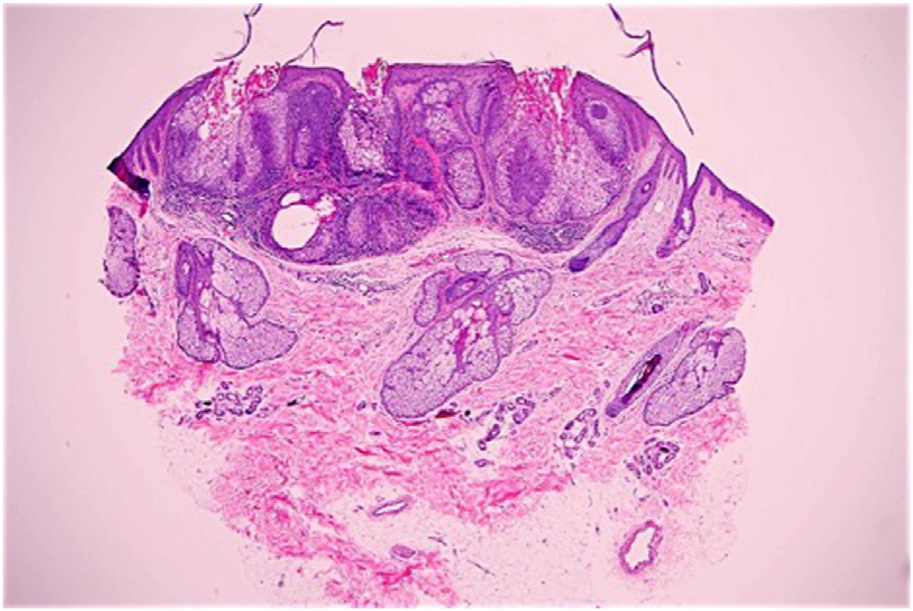
Original magnification 40×. Histologic sections demonstrate a sharply demarcated, endophytic lobular growth of sebocytes, and basaloid germinative cells arising directly from the epidermal undersurface.

A 52-year-old woman with a medical history of endometrial carcinoma treated with chemotherapy and hysterectomy, MTS, and 17 nonmelanoma skin cancers – which included 11 sebaceous adenomas and 2 sebaceous hyperplasias as well as other basaloid epithelial neoplasms, and squamous cell carcinomas – who presented for concerns of multiple facial lesions. A punch biopsy revealed a sebaceous adenoma (Fig 1). Given her history of MTS and the development of new sebaceous neoplasms, she was referred to gastroenterology where a rectal carcinoma was identified via colonoscopy. Our patient underwent a total proctocolectomy and continued to follow with dermatology regularly. Four years later, she developed 2 sebaceous adenomas that arose synchronously with premalignant gastric polyps and a subsequent endoscopy identified a gastric adenocarcinoma. Given concerns regarding the patient’s quality of life, a partial gastrectomy was performed and acitretin was initiated as a chemoprophylactic agent for her gastrointestinal carcinomas and sebaceous tumors. Prior to acitretin therapy, atorvastatin was started due to the patient’s hypercholesterolemia with noted resolution of her lipid abnormalities. Acitretin 10 mg daily was started and titrated up to 25 mg daily with clearance of the patient’s facial lesions and serial workup of her liver function and lipid panels were within normal limits. After several months of therapy, acitretin was decreased to 10 mg daily due to medication side effects. At 10 mg per day, she developed 5 sebaceous tumors, including one sebaceous carcinoma and one atypical cystic neoplasm with sebaceous differentiation; other than these sebaceous tumors, our patient did not develop any other nonmelanoma skin cancers while on acitretin therapy. These neoplasms were surgically excised and the patient’s acitretin was increased to 17.5 mg daily. Her cutaneous findings have remained clinically stable at this dose for 5 years. In almost 11 years of acitretin therapy, our patient’s visceral malignancies have shown no evidence of recurrence. In addition, our patient discontinued her atorvastatin and maintained stable lipid and liver function panels while on therapy.

## Discussion

Clearance and or decrease in sebaceous neoplasm sizes have been described in three MTS patients on isotretinoin and one patient on acitretin.^1^, ^2^, ^3^, ^4^ Upon treatment cessation, recurrence of sebaceous neoplasms and development of a new squamous cell carcinoma were reported (Table I). We report a similar case where clearance of sebaceous neoplasms was observed while on acitretin therapy and recurrence was noted upon decreasing the patient’s acitretin dose. This suggests a dose-dependent response and need for long-term adherence for continued chemoprophylaxis with oral retinoids.

**Table I:** Cases of retinoid treatment and response for cutaneous and visceral malignancies

Ref	Age, sex	Treatment duration	Therapy	Cutaneous response to treatment	Effect of discontinuation	Visceral malignancies	Visceral malignancy response while on treatment
1	51F	28 wk	Isotretinoin	- Reduction of sebaceous neoplasms - Clearance of KA	- Sebaceous neoplasm recurrence	- Family history positive for visceral malignancies	- Not reported
	56M	14 mo	Isotretinoin	- Reduction of sebaceous neoplasms - No new epithelial tumors	- Not reported	- Transitional cell carcinoma of kidney	- Not reported
2	57M	12 mo	Isotretinoin + IFN-a2a	- Reduction of sebaceous neoplasms - 1 new skin tumor in 29 mo	- Sebaceous neoplasm recurrence	- Transitional cell carcinoma of kidney, adenoma of colon	- No internal tumor recurrence
3	55M	Not reported	Isotretinoin + CO2 Laser	- Reduction of sebaceous neoplasms - Clearance of most sebaceous neoplasms	- Sebaceous neoplasm recurrence	- Adenocarcinoma of colon	- Not reported
4	49M	12 y	Acitretin	- Reduction of sebaceous neoplasms - Four new cutaneous lesions	- New SCC	- Benign pancreatic tumor, benign adenomatous colonic polyps, dysplastic tubulovillous adenomas	- No internal tumor recurrence
Current case	56F	11 y	Acitretin	- Clearance of sebaceous lesions within 3 mo	- Sebaceous neoplasm recurrence - New sebaceous carcinoma	- Gastric adenocarcinoma, uterine carcinoma, rectal carcinoma	- No internal tumor recurrence

*KA*, Keratoacanthoma; *SCC*, squamous cell carcinoma.

The gastric literature has reported expression of retinoid receptors in colorectal carcinomas.^5^ This may explain how retinoids exert their chemoprotective effects in both organ systems, as observed in our patient and in previously reported cases (Table I).^1^, ^2^, ^3^, ^4^ The gastric literature also describes defects in vitamin A metabolism in colorectal carcinomas. Multiple mechanisms have been proposed for how retinoids inhibit tumorigenesis. A study led by Penny et al reported retinoic acid reducing tumor burden in patients with familial adenomatous polyposis by preferentially inducing regulator T-cells.^6^ Further research is warranted to elucidate the mechanism of action of retinoids in the gastrointestinal tract.

Our case documents longitudinal chemoprophylactic management with acitretin for both sebaceous and gastrointestinal neoplasms in a patient with MTS. Given the development of new sebaceous neoplasms when our patient’s acitretin dose was lowered, this supports prior reports that the retinoid’s therapeutic effects may be dose-dependent for cutaneous malignancies seen in MTS. This approach has minimized surgery for new cutaneous neoplasms and averted a total gastrectomy, which would have had a significant negative impact on her quality of life. Additional research is needed to explore optimal treatment dose and duration for chemoprevention of cutaneous and visceral malignancies observed in MTS.

## Conflicts of interest

None disclosed.
